# Biomechanical Comparison of Medio-Plantar and Plantar Plate Fixation for First Tarsometatarsal Joint Arthrodesis

**DOI:** 10.3390/jcm12123896

**Published:** 2023-06-07

**Authors:** Kajetan Klos, Paul Simons, Pauline Schopp, Philipp Schenk, Felix C. Kohler, Akram Uddin, Edgar K. Roth, Uta Biedermann, Gunther O. Hofmann, Mark Lenz

**Affiliations:** 1 Department of Trauma, Hand and Reconstructive Surgery, Jena University Hospital, Friedrich Schiller University Jena, 07747 Jena, Germany; 2 Foot and Ankle Division, Gelenkzentrum Rhein-Main, Frankfurter Str. 94, 65239 Hochheim am Main, Germany; 3 Foot and Ankle Division, St. Josefs-Hospital Rheingau, Eibinger Str. 9, 65385 Rüdesheim am Rhein, Germany; 4 Research Executive Department, BG Klinikum Bergmannstrost, 06112 Halle, Germany; 5 Department of Podiatric Surgery, Northamptonshire Healthcare NHS Foundation Trust, Danetre Hospital, London Road, Northamptonshire NN11 4DY, UK; 6 Department of Podiatric Surgery, Essex Partnership University NHS Foundation Trust, Rochford Hospital, Essex, Rochford SS4 1RB, UK; 7 Institute of Anatomy I, Jena University Hospital, Friedrich Schiller University Jena, 07743 Jena, Germany

**Keywords:** Lapidus bunionectomy, bunion, hallux abducto valgus, locking plate

## Abstract

Plantar plate positioning has been demonstrated as biomechanically superior. However, some operators remain resentful about the morbidity of the surgical approach. To provide improved plate fixation for first tarsometatarsal joint arthrodesis with respect to the tibialis anterior tendon, a medio-plantar plate was developed. The purpose of this biomechanical study was to compare its construct stability to that of a plantar plate construct. Twelve pairs of fresh frozen human specimens were used in a matched pair test. Each pair was fixed with a 4 mm compression screw and either a plantar locking plate or a medio-plantar locking plate. A cantilever beam test was performed in dorsiflexion. Before and after cyclic loading (5000 cycles; 40 N), bending stiffness and relative movements at the joint space were monitored in a quasi-static test including optical motion tracking. Maximum load and bending moment to failure were investigated in a load-to-failure ramp test. The bending stiffness of both groups did not significantly differ before (plantar 49.9 N/mm ± 19.2; medio-plantar 53.9 N/mm ± 25.4, *p* = 0.43) or after (plantar 24.4 N/mm ± 9.7; medio-plantar 35.3 N/mm ± 22.0, *p* = 0.08) cyclic loading but decreased significantly in both groups (*p* < 0.01) after cyclic loading. Relative movement increased significantly during cyclic testing in both groups (*p* < 0.01) but did not differ significantly between the groups before (*p* = 0.29) or after (*p* = 0.16) cyclic loading. Neither load nor bending moment to failure were significantly different (plantar 225 N ± 78, 10.8 Nm; medio-plantar 210 N ± 86, 10.1 Nm, *p* = 0.61). Both plate constructs provided equivalent construct stability, both being well suited for Lapidus arthrodesis.

## 1. Introduction

Paul Lapidus described tarsometatarsal (TMT) joint arthrodesis for the treatment of severe hallux valgus deformity in 1934 and became the eponym of this technique [1,2]. However, the procedure itself was described in German literature as early as 1911 by Albrecht [3]. However, since sufficient possibilities of stabilization were lacking, the technique only really became common with the introduction of modern implants in orthopedic surgery [4]. In modern practice, arthrodesis of the first tarsometatarsal joint alone as a modification of Lapidus arthrodesis is a widely used procedure for the correction of hallux valgus deformity associated with instability of the first TMT [4,5,6,7]. 

The introduction of angle-stable implants should lead to earlier weight-bearing and a higher union rate [7]. Biomechanical studies showed, however, that a dorso-medial plate alone was inferior to a two-screw arthrodesis in a purely static test [8]. An equivalent stability between a dorso-medial plate and a two-screw arthrodesis could only be demonstrated in combination of a dorso-medial plate with a compression screw [9]. Both results reflect the importance of a compression screw in arthrodesis of the first TMT, corresponding to the Arbeitsgemeinschaft für Osteosynthesefragen (AO) principle of combining a compression screw and neutralization plate [10]. In further biomechanical studies using a cyclic loading protocol, the superiority of a medial plate with compression screw over a crossed two-screw technique was proven [11]. Thus, it could be shown that turning the plate position towards the bending side improves stability. Furthermore, another biomechanical study showed the superiority of a plantar plate in comparison with a dorso-medial plate [12]. Thus, the AO principle of tension banding was again proven [10]. This biomechanical advantage of plantar plate positioning compared with other plate positioning and the use of large intramedullary devices was confirmed by several independent working groups [12,13,14,15].

The advantages of plantar plate positioning have also been confirmed in several, partly comparative, independent clinical studies [16,17,18,19,20]. For example, the biomechanical advantages mean that, although patients are allowed protected full weight-bearing immediately postoperatively, the risk of nonunion reported in the literature is only 1% [16,17,18,19,20]. Thus, plantar plate positioning has now established itself as a standard method in many countries. However, some operators remain resentful about the morbidity of the surgical approach. Although these concerns were refuted in an anatomical study, [21], Gutteck et al. were able to show in a clinical study that the medial approach improves the reduction of the first metatarsal with respect to elevation, compared with a dorsal approach [19]. Nevertheless, there is still the fear of an injury to the tibialis anterior (TA) tendon. Although this has been described for various plantar plate systems in anatomical studies, clinically relevant injuries to the TA tendon are reported anecdotally at best and have not been published in any clinical study [19,20,21,22,23,24,25,26,27]. In the most well-founded publication on this subject, Niehaus et al. presented a study using magnetic resonance imaging (MRI) after Lapidus arthrodesis with one plantar plating system to identify tendinopathy of the tibialis anterior (TA) tendon [27]. They found signs of tendinopathy in more than half of the cases but did not see any effect on the clinical outcome [27]. The researchers in that study concluded that injuries of the TA tendon must be considered when choosing the desired implant and placement [27].

Medial plate positioning continues to be thought of as a safe alternative, even if this can also be proven to cause irritation of the TA tendon [21]. Thus, it seems only logical to combine the advantages of both methods to avoid TA tendon injuries without having to forego other advantages.

However, since a new plating system must also prove itself biomechanically, we investigated a new medio-plantar plate construct in comparison with a plantar plate.

As a null hypothesis, comparable stability of both constructs was formulated.

## 2. Materials and Methods

### 2.1. Specimens

Twelve fresh frozen (−20 °C) pairs of foot specimens were harvested, from 10 male and 2 female donors (mean age, 83.5 years; range, 67 to 91 years). Conventional anteroposterior and mediolateral radiographs were taken from all specimens to confirm absence of preexisting pathology or fractures. The specimens were thawed at room temperature 24 h prior to instrumentation, embedding, and mechanical testing. Left and right specimens were assigned randomly to either group (plantar or medio-plantar), allowing a pairwise comparison and ensuring that each study group contained an equal number of right and left specimens.

The first metatarsal, the medial cuneiform, and the navicular bone were removed en bloc, with preservation of the ligaments and the joint capsules; the footprint of the TA tendon was kept in situ, to guide plate positioning. The joint surfaces were preserved; the capsular and ligamentous structures of the metatarso-cuneiform joint were resected after instrumentation.

### 2.2. Instrumentation

**Figure 1:**
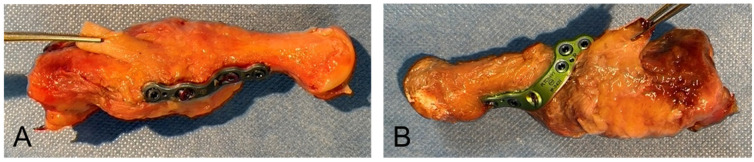
Instrumented specimens with left plantar plate (**A**) and right medio-plantar plate (**B**). Tibialis anterior tendon is elevated.

Instrumentation was performed by two experienced foot surgeons (P.S. and K.K.). All implants were of titanium alloy. Plate design differed between the groups. The screws were of the same type and diameter (Figure 1). Appropriate implant positioning was confirmed by conventional radiography after instrumentation.

First, a 4 mm diameter cannulated self-tapping partially threaded compression screw (QLT3.5Lxx, Newclip Techniques, Haute-Goulaine, France) was inserted into all specimens. Screw placement was performed as described by Gruber et al. [9] and Baravarian et al. [28]. For screw positioning, a guidewire was placed across the 1st TMT joint from the distal dorso-medial aspect of the 1st metatarsal at a distance of 10 to 15 mm from the joint space to the proximal plantar lateral aspect of the medial cuneiform and over drilled. The screw was introduced via the guidewire. Compression was achieved by screw tightening using the three-finger technique. All screws were inserted with an appropriate length for bicortical screw purchase.

### 2.3. Plantar Plate Construct

At the plantar aspect of the 1st TMT joint, an anatomically pre-shaped side specific initial FTM—Lapidus 3.5 mm plantar Lapidus arthrodesis plate (size 1, right: FLTDV1, left: FLTGV1, Newclip Technics, Haute-Goulaine, France) was placed.

Following the manufacturer’s instructions, 3.5 mm non-locking screws were inserted into the inner screw holes, to obtain additional compression. Into the outer screw holes, 3.5 mm fixed-angle locking screws were placed.

### 2.4. Medio-Plantar Plate Construct

At the medio-plantar aspect of the 1st TMT joint plantarly from the metatarsal I to the medial aspect of the medial cuneiform, an anatomically pre-shaped side-specific initial FTM—Lapidus 3.5 mm plantar Lapidus arthrodesis plate (size 1, right: FLTDMV1, left: FLTGMV1, Newclip Technics, Haute-Goulaine, France) was placed. Into the central screw hole and the inner screw holes at each side, 3.5 mm nonlocking screws were inserted to obtain additional compression. Into the outer screw holes, one at the 1st metatarsal and two at the medial cuneiform, 3.5 mm fixed-angle locking screws were placed.

### 2.5. Potting

The embedding was performed as described previously [14]. Prior to potting, the navicular bone was fixed to the medial cuneiform bone with three Kirschner wires to enlarge the surface available for potting. Correct Kirschner wire positioning without compromising the screws or crossing the tarsometatarsal joint space was verified radiographically. The ligaments and the joint capsules of the 1st TMT joint were resected. All exposed implant surfaces were covered with plasticine to prevent direct contact with the polymethylmethacrylate. For embedding, each specimen was aligned in a potting fixture along its longitudinal axis from the center of the head of the 1st metatarsal to the center of the proximal joint surface of the navicular. The navicular and the medial cuneiform were placed into a 55 mm diameter metal tube and potted in polymethylethacrylate (PMMA, Technovit^®^, Kulzer GmbH, Wehrheim, Germany). The 1st TMT joint remained outside the PMMA cylinder. The distal end of the 1st metatarsal was potted in a 27/30 mm diameter PMMA cylinder so that a distance of 48 mm between the two PMMA cylinders including the 1st TMT joint remained unembedded.

### 2.6. Mechanical Testing

**Figure 2:**
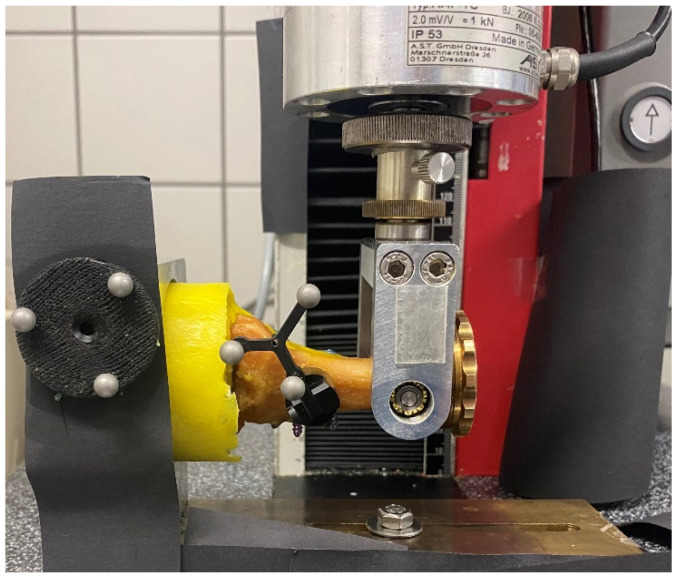
Test setup with potted specimen fixed on the left as cantilever beam and connected to the load cell via a hinge. Two retroreflective marker sets for optical motion tracking are attached to the fixture (left) and to the 1st metatarsal shaft (right).

A material testing machine (Zwick 1.0; Zwick GmbH, Ulm, Germany) with a 1 kN load cell operated in load control mode was used for testing. Load and displacement were recorded from the test system’s transducers at a rate of 10 Hz. Test setup and load protocol were considered reasonable according to pretests and have been used by other authors in a similar test setup [13,14]. Specimens were fixed in the test setup bottom-up in a dorso-plantar position ensuring a physiological load bearing of the construct with bending moment as the main load. Mechanical testing was performed via a cantilever beam bending test with loading in tension and compression. Load was applied on the distal end of the metatarsal I (Figure 2). Specimens were kept moist during the test procedure.

Before and after cyclic testing, a quasi-static bending test of ten cycles (plus three conditioning cycles) was performed, with a load ramp from −5 to +20 N at a cross-head speed of 0.5 mm/s. During quasi-static bending testing, relative movements at the joint space were measured via monitoring of two retro-reflective marker sets fixed at the metatarsal I shaft and the fixture of the proximal PMMA cylinder, by means of optical motion tracking with two digital cameras (kolibri CORDLESS; measurement uncertainty of 20–100 µm; Fraunhofer Institute for Applied Photonics and Precision Engineering IOF; Jena, Germany) at a rate of 50 Hz. Cyclic loading was performed over 5000 load cycles with rectangular load from −5 to +40 N at a cross-head speed of 2 mm/s. Finally, the specimens were loaded in dorsiflexion up to failure with a load ramp starting from 0 N at a cross-head speed of 0.5 mm/s.

### 2.7. Data Analysis

Bending stiffness before and after cyclic testing was determined during the quasi-static test according to the gradient of the load–displacement curve in the quasilinear elastic region. Relative movements at the joint space before and after cyclic testing were calculated from the machine data and optical data of the quasi-static test. Load to failure was determined from the displacement progression during the ramp test. Failure was defined as a sudden drop in the load–displacement curve. Machine data were evaluated by means of Zwick testXPert III software, (Zwick GmbH, Ulm, Germany). Optical data were evaluated using Matlab software package (R2019b; The Mathworks, Natick, MA, USA).

Statistical analysis was performed with the use of SPSS software package (IBM SPSS Statistics 27; SPSS Inc., Chicago, IL, USA). Normality of data distribution within each group was verified by the Shapiro–Wilk test. Paired samples *t*-testing and the Wilcoxon signed-rank test were applied to indicate significant differences between the two groups regarding bending stiffness, relative movement, and failure load. Significance level was set at 0.05 for all statistical tests.

## 3. Results

Bending stiffness of both groups did not differ significantly before (*p* = 0.43) and after (*p* = 0.08) cyclic loading. Bending stiffness decreased significantly in both groups after cyclic loading compared with the initial test, see Table 1.

**Figure 3:**
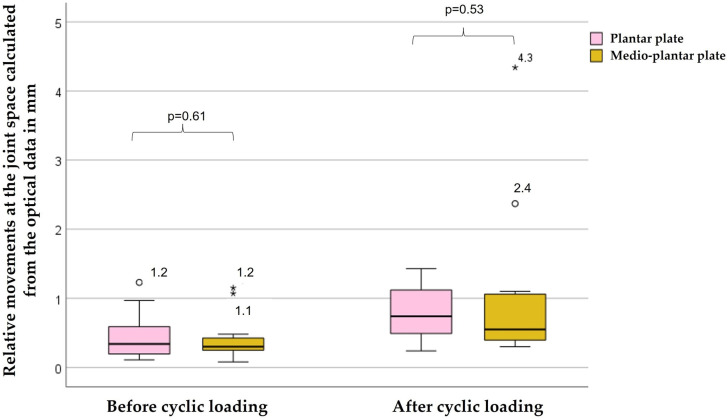
Relative movements at the joint space calculated from the optical data in mm. The box indicates the median with interquartile range. Whiskers represent the range at greatest length 1.5 times the interquartile range. Outliers are marked separately. Circles mark outliers within the 10% quartile, asterisks mark outliers over 3-fold interquartile range. Differences in relative movements between the groups before (*p* = 0.61) and after (*p* = 0.53) cyclic loading were not significant.

Relative movements at the joint space calculated from the optical data are shown in Figure 3. Differences in relative movements between the groups before (*p* = 0.61) and after (*p* = 0.53) cyclic loading were not significant. Relative movements increased significantly in the plantar group (*p* < 0.01) and the medio-plantar group (*p* < 0.01) after cyclic loading compared with the initial test.

Relative movements at the joint space calculated from the machine data are given in Table 2.

Mean load and corresponding bending moment to failure did not differ significantly between the plantar group (mean 225 N ± 78; mean 10.8 Nm) and the medio-plantar group (210 N ± 86; mean 10.1 Nm), *p* = 0.61. In both groups, total construct failure occurred in bending at the joint with plantar opening at the joint space.

## 4. Discussion

For a long time, screw fixation was the most common technique for Lapidus arthrodesis and remains an acceptable procedure [5,28,29]. However, with the development of special angle-stable plating systems, the use of plates is playing an increasingly important role [7].

As early as 1998, Marks et al. [30] showed in a biomechanical study on metatarso-cuneiform fusions that a strictly plantar one-third tube plate provides superior stability compared with screw arthrodesis. In this respect, a positive biomechanical effect of plate positioning towards the tension side could already be concluded at that time. Following this AO principle [10], modern plantar plate systems were developed whose biomechanical superiority compared with other plate positioning and intramedullary methods could then also be demonstrated by independent authors [14,15,16,17,18]. The advantages of plantar plate positioning have also been confirmed in several independent clinical studies [19,20,22,23,24]. Thus, from our point of view, new implant systems for modified Lapidus arthrodesis must be able to be measured on a plantar plate.

In the present study, an anatomically shaped medio-plantar plate developed for first TMT joint arthrodesis was selected for testing. The plate has been designed in such a way that it adapts well to the contour of the joint and thus avoids protrusion, which could lead to soft tissue irritation. This plate is shaped in such a way that damage to the TA tendon should be avoided, and the approach can be kept smaller than with a plantar plate arthrodesis, as less of the plantar aspect of the first ray has to be prepared. Better stability should be given by the partially plantar plate orientation compared with a medial plate, which is also more biomechanically advantageous than a screw arthrodesis [11,12].

A system that was already available locally was chosen as the comparison implant. The implant was chosen as it has almost the same length as the medio-plantar plate, uses the same screws and locking system, and is constructed from the same titanium alloy. We would expect better performance with three screws on each side of the plantar plate. However, this plate has only four instead of six screw holes, which we consider to have possible biomechanical disadvantages.

None of the biomechanical parameters we examined showed a difference in the comparison of the implants. However, there was a significant difference within the two groups before and after cyclic testing. This expected difference shows that the test setup was suitable for this purpose. The results of the measurement of the relative movements obtained from the optical system coincide with the machine data, which underlines the reliability of our measurements.

With our results, we were able to confirm our hypothesis that a medio-plantar angle-stable plate with compression screw and the currently common plantar angle-stable plate with compression screw provide comparable mechanical stability.

A medio-plantar plate can also have disadvantages compared with plantar plates. For example, we know that the use of a medial plate causes more soft tissue irritation than a plantar plate [19]. This disadvantage is also conceivable with the medio-plantar plate and must be investigated further in clinical and anatomical studies. In common with all biomechanical investigations, we identified that our study had certain limitations. In particular, bone consolidation and the influence of soft tissue were not addressed. Following the study by Marks et al. [30] and other studies on arthrodesis, the articular surface was left [11,13,31,32]. Thus, the biomechanical influence of various preparations of the articular surfaces as shown by Ray et al. could be eliminated [33]. This has an influence on the biomechanics, due to the resulting different positioning and therefore fitting of the implants. Furthermore, the capsule and all ligaments were removed to create a standardized worst-case scenario. The donor age was very high with an average of 83.5 years, and more male than female donors were included. Thus, the group does not correspond to the expected average patient population, but reflects the high-risk patient who requires particularly stable care [6,7]. The number of cycles was also based on Marks et al. [30]; however, this would not equate to the presumed stresses on the construct during the time to sound bony union, especially where immediate postoperative full weight-bearing is allowed. The subsequent loading to failure represented a worst-case scenario of a fall or a missed footing, rather than the repetitive loading and unloading of the construct over a period of several weeks, which constitutes the main cause of nonunion and subsequent implant failure observed in actual patients [8,9,12]. The results of the present study thus cannot be directly translated into the clinical setting.

## 5. Conclusions

Both plates showed no biomechanical difference. These results encourage clinicians to allow protected full weight-bearing immediately postoperatively in patients who are treated with a medio-plantar plate. Whether this biomechanical equivalence combined with the novel design of the medio-plantar plate is then reflected in reduced irritation of the TA tendon requires further investigation. Anatomical and clinical studies are therefore required on the outcomes of subjects undergoing Lapidus arthrodesis with the medio-plantar plate described in this study.

## Figures and Tables

**Table 1:** Mean bending stiffness values with standard deviation (SD) of the plantar and medio-plantar group at the beginning and after 5000 cycles are shown including the *p* values of the comparisons between the time points (0 cycles, 5000 cycles) within each group. * Significant difference.

Bending Stiffness	Plantar		Medio-Plantar	
(N/mm)	0 Cycles	5000 Cycles	0 Cycles	5000 Cycles
Mean	49.9	24.4	53.9	35.3
SD	19.2	9.7	25.4	22.0
*p* value	0.01 *		0.01 *	

**Table 2:** Mean relative movement values at the joint space with standard deviation (SD) calculated from the machine data of the plantar and medio-plantar group at the beginning and after 5000 cycles are shown including the *p* values of the comparisons between the time points (0 cycles, 5000 cycles) within each group and between the groups at the different time points. * Significant difference.

Relative Movement	Plantar		Medio-Plantar	
(mm)	0 Cycles	5000 Cycles	0 Cycles	5000 Cycles
Mean	0.7	1.4	0.6	1.3
SD	0.2	0.5	0.1	1.0
*p* value	0.01 *		0.01 *	
plantar–medio-plantar	0 cycles		5000 cycles	
*p* value	0.29		0.16	

## Data Availability

Not applicable.
